# Supplementary material for: The Chromosomal Distribution of Sex-Biased MicroRNAs in Drosophila is Nonadaptive
Source: Genome Biol Evol. 2022 Jul 9;14(7):evac103. doi: 10.1093/gbe/evac103 (PMC9290354; doi:10.1093/gbe/evac103)
Supplement: evac103_Supplementary_Data [file evac103_supplementary_data.zip › Supplementary_figure_1.pdf]

Supplementary Figure 1

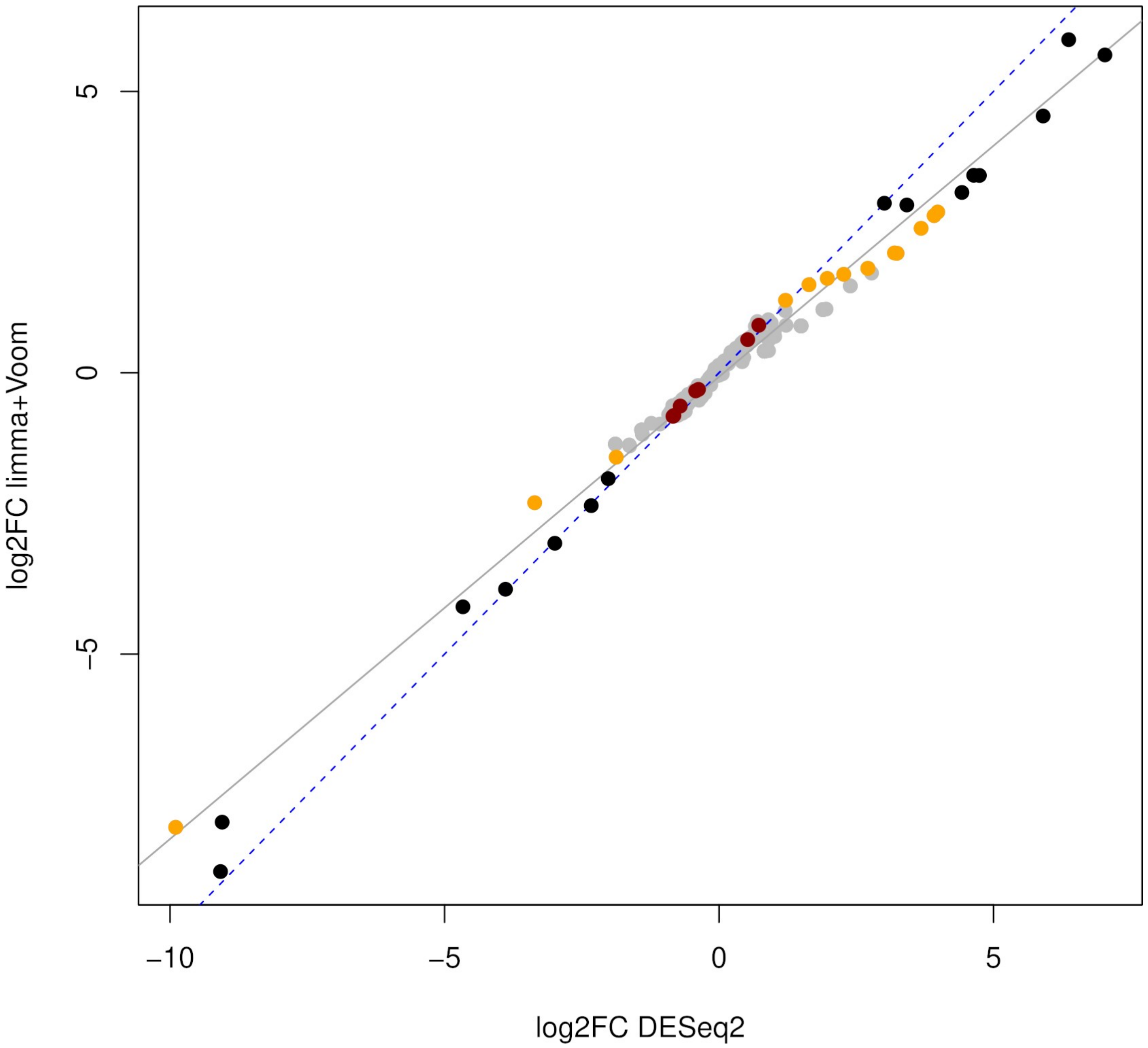

Supplementary Figure 1. Comparison between the log2 fold-changes from the differential microRNA expression analysis of the paired samples with DESeq2 and Limma with Voom transformation. Dot color code: black, differentially expressed (DE) according to both methods; orange, DE according to Voom-Limma only; red, DE according to DESeq2 only; grey, not DE with either method.
